# Selections

**Published:** 1875-04

**Authors:** 


### Selections.

### ON THE ALLEGED SUCCESSFUL TREATMENT OF TYPHOID FEVER BY "COLD."

BY FREDERICK T. ROBERTS, M.D., B.Sc., M.R.C.P.,

Assistant Physician and Teacher of Clinical Medicine at University College Hospital, etc.

Among the remedial measures which are attracting attention at the present time, some of the most important are those which have come into prominence in connection with the treatment of febrile diseases, and which have for their object the application of cold to the external surface of the body. Of course they do not involve any newly-discovered principle, for they are merely revived methods of a plan of treatment which is more particularly associated with the name of Currie, who laid down definite rules as to how and when it should be carried out. These measures have been during recent years much more extensively and generally employed on the continent, and especially in Germany, than in this country; but anyone who is acquainted with current medical literature must be familiar with the fact that they have not been ignored by the profession here, but have been duly recognized as affording valuable aid in the management of fevers, under certain circumstances, and that their efficacy has been tested by several accomplished observers, such as Wilson Fox, H. Weber, Ringer, Thompson, Greenhow, Clifford Allbutt, and others.

The object of the present paper is to offer a few remarks with reference to the alleged advantages of the use of "cold" in typhoid fever. But before considering the matter in connection with this particular complaint, I wish to make some observations of a general character. First, most of the methods by which the application of cold is effected require much care and attention in carrying them out; they must be conducted under the personal supervision of a competent medical attendant, and their effects need to be thoroughly watched. They have been lately alluded to as "simple remedial measures," but this they are not in any sense, for most of them involve no small difficulty in their execution, especially in private practice, while as regards their effects on the system, they by no means come under the category of simple, harmless measures, such as may be adopted in a routine, off-hand fashion. Secondly, the more evident effects which have been noted from the employment of these applications in febrile cases are, the reduction of the temperature of the body; diminution in the frequency of the pulse; the checking of tissue change, as evidenced by the excretions and by wasting being less rapid; the relief of nervous symptoms; and the modification of the eruption in certain of the exanthemata. The eruption may be encouraged to come out, as in scarlatina or measles; or, it is stated, it may be diminished in amount and improved in quality, as in small-pox. It is in the treatment of cases attended with very high temperature—cases of hyperpyrexia—that the beneficial results of applying cold to the surface, by means of baths, etc., have been most signally obtained; and here it may be said, without any exaggeration, to have saved the lives of patients who must inevitably have died had not this method of treatment been adopted.

Several German physicians have spoken highly of the treatment of ordinary cases of various fevers, and among them of typhoid fever, to which disease I desire now to call attention more particularly. In a paper on typhoid fever in the recent volume of the St. Bartholomew's Hospital Reports, Dr. Gee considers the use of external cold in its treatment, but apparently rather from a hypothetical point of view than from any actual experience of its advantages. He implies that, if employed at an early period, during the primary fever, it might control the local intestinal lesion, by limiting the number of follicles involved; while in a more advanced stage it might be useful in reducing the secondary fever which accompanies the morbid changes in their progress.

The conclusions of those observers who have practically tested this method of treatment deserve our thoughtful attention, and it is important to ascertain what they affirm respecting it. None of those who are reliable pretend that they can actually *cure* typhoid fever, either in the sense of checking the disease at the outset, or even of shortening its course. To talk of *curing* or *arresting* this complaint shows a want of knowledge as to its nature. At the best, we can only guide it in its progress, and endeavor to bring the patient successfully through the dangers which he has to encounter. This remark applies not only to the treatment now under consideration, but to any other which claims an influence of this kind over typhoid. Some believe that the convalescence is more rapid after the treatment by cold; but others deny this. So also is there a difference of opinion as to whether it affects the more prominent symptoms, such as diarrhœa, tympanitis, intestinal hæmorrhage, etc.; some asserting that these are beneficially influenced, others that they are not. All seem to agree that it does not increase the tendency to lung-inflammations, or aggravate these if they already exist. The most important statement made is, that this method of treatment has greatly reduced the mortality from typhoid fever, particularly when the cases have been treated from an early period. Certainly the returns made show a great difference in this respect, when the past is compared with the present, and seem to indicate a highly satisfactory result. At the same time, before any certain conclusion can be arrived at, a large number of cases ought to be taken into consideration; for it often happens that a series come under treatment which are of a mild type, and the mortality may present a very low percentage, whatever treatment may have been adopted; while some epidemics are very fatal. Judging from personal experience, and combining hospital with private practice, I believe that the rate of mortality has not been higher in the cases which have come under my own notice than it is represented in the German returns. The mortality in former times was decidedly high, and it would be well to know what modes of treatment were then adopted, as they were not always of a harmless character in days gone by, while the diet and hygienic conditions—most important elements in the treatment of typhoid—were not attended to then as they are now.

Those who adopt this plan of treatment are anything but agreed as to the details of carrying it out. The principal methods employed are frequent cold or tepid sponging of the skin, wet-packing, cold baths, affusion, and tepid baths gradually cooled. Some pursue the treatment vigorously in all cases; others only when the temperature reaches a certain height. There are also differences as regards the duration of each application, the frequency of their repetition, and the length of time during which they are continued.

I venture to state briefly the conclusions which I have arrived at with respect to the treatment now under consideration.

1. It is highly desirable that the members of our profession should be more generally impressed than they are at present, with the usefulness of the various modes of applying cold to the surface of the body in febrile cases, under certain circumstances; and that they should be prepared without hesitation to carry one or other of them out efficiently whenever this plan of treatment is indicated. This applies to typhoid in common with other fevers.

2. On the other hand, to adopt a routine hydropathic treatment of any fever seems to me most objectionable, and this applies especially to the more severe methods which are advocated. As already remarked, they are not easily carried out in general practice; they are certainly not required in a large proportion of cases; most of them are anything but pleasant to the patients, and they may prove very trying and exhausting, especially if frequently repeated, as they usually need to be if the treatment is efficiently fulfilled; while it must be remembered that they are not harmless measures, but may have a powerful influence for evil as well as for good. With regard to typhoid, many cases do not come under observation until it is too late to attempt to check the primary fever, even supposing that the intestinal lesion could be thus limited. For these and other reasons I do not see that, at present at least, a hydropathic treatment of typhoid fever in general practice has any claim to our support. If it is thought worthy of trial, it ought first to be fairly tested in *bona fide* cases of this disease, and under the strictest and most competent supervision. With regard to sponging of the skin, I believe that this is often very useful, and ought to be employed far more frequently than it is at present, in typhoid as well as in other fevers. With proper care it does no harm, while it often gives much relief, and is beneficial in other respects.

3. The cases in which the more severe methods of applying cold are indicated are those in which the temperature is already very high and remains so, or shows a tendency to rise rapidly, especially if at the same time there are signs of much nervous disturbance. Unquestionably this plan of treatment is not resorted to under these circumstances nearly so frequently as it ought to be. It is difficult to lay down any exact rule as to what temperature indicates the necessity for adopting it, but if it reaches to 106° F. and shows no tendency to fall, or, still more, if it continues to rise, this treatment deserves due consideration. Necessarily much will depend on the actual condition of the patient, and every case must be thoroughly considered in all its features. The best method seems to me decidedly that of placing the patient in a tepid bath, and gradually cooling this. Affusion over the head is useful if there are marked nervous symptoms. Of course it is imperative that this treatment should be always conducted under the strictest supervision, and its effects carefully watched.—*Practitioner.*

---

### ON THE PHYSIOLOGY OF THE ACT OF VOMITING.

BY W. H. BROADBENT, M.D., F.R.C.P.

In the interesting article on vomiting in the *Practitioner* for January, by the Editor, an active part in the expulsion of the contents of the stomach is attributed to the diaphragm ; that is, the stomach is said to be compressed by a simultaneous contraction of the diaphragm and abdominal muscles. This, moreover, is what is taught in most of the standard works on physiology, and it is considered to be established by an experiment in which the contents of the stomach were expelled by the contraction of the diaphragm after the abdominal parietes had been cut away to near the linea alba.

We cannot attach demonstrative value to an experiment in which such extensive mutilation is practiced, and I have been led by observation of the phenomena of vomiting to question the accuracy of the generally received account of its production, and to come to the conclusion that the contraction of the diaphragm does not coincide in time with that of the abdominal muscles, but that the diaphragm first descends and is fixed by closure of the glottis, forming thus a firm but passive and merely resisting plane, against which the stomach is pressed by the actively contracting muscles of the abdominal wall.

I might cite in favor of this view the anatomical consideration that the œsophageal opening in the diaphragm lies between the decussating fibres of the pillars, and that when these fibres are contracting powerfully they will compress the œsophagus. This point, however, has frequently been set forth, and all *a priori* arguments, even from anatomical facts, must yield to actual observation.

I have attempted direct experimental observations, but under circumstances which did not permit of definite conclusions, since they were made on animals in which the anterior wall of the thorax was removed for the purposes of another investigation. Such as they were, however, they were confirmatory. But I must rely entirely on what may be noted in the human subject in the act of vomiting. During the antecedent nausea and in the intervals of the paroxysms, there is shallow respiration with a wide-open glottis, together with frequent sighing. A paroxysm is ushered in by a sudden inspiratory gasp—that is, by a descent or contraction of the diaphragm—and the success of the effort of expulsion appears to depend greatly upon the extent of this descent, and upon the point at which the diaphragm is arrested and fixed by the closure of the glottis. If the diaphragm were in active contraction at the moment of actual vomiting, we might expect that at any rate some of the sounds then produced would be inspiratory, whereas they are entirely expiratory, and much air is often forced out of the chest by the same act which expels the contents of the stomach. Any contraction of the diaphragm must thus be overcome as well as the resistance of the glottis. Another consequence of contraction of the diaphragm as a part of the expulsive effort in vomiting would be a tendency to inspiration of the vomited matters while passing over the orifice of the larynx, which we might expect to see abundantly realized from time to time; whereas any slight entry of these matters which takes place, occurs in the inspiration which follows the actual vomiting. This same deep inspiration, again, after the convulsive retch, would appear to be conclusive evidence that the diaphragm had no share in it. It may be added that we should scarcely have the powerful compression of the lungs and heart in the act of vomiting, of which we sometimes seek to avail ourselves therapeutically, were the diaphragm contracting entirely against the expiratory muscles. Finally, the expulsive effort may be defeated by maintaining the glottis open. Such, at least, is my conclusion from a painful personal experience or experiment carried to the extent of my powers of endurance.

This note may be fitly ended by an observation in which the theory of the act of vomiting here maintained was turned to practical account. While resident medical officer to St. Mary's Hospital I performed the operation of tracheotomy on a young child. Two or three days afterwards I was called to the child, and found it in convulsions. Watching it carefully, I perceived that the general convulsive movements were preceded by violent contraction of the abdominal muscles, with facial movements suggestive of vomiting. The attempt to expel the contents of the stomach was, however, defeated by the absence of *appui* for the diaphragm by closure of the glottis, due to the opening in the trachea. Recognizing this, I closed the tube at the moment of each expulsive effort; free vomiting occurred, and the convulsions at once ceased.—*Practitioner.*

